# Impact of metabolic syndrome on lipid target achievements in the Arabian Gulf: findings from the CEPHEUS study

**DOI:** 10.1186/s13098-016-0160-6

**Published:** 2016-07-26

**Authors:** Ibrahim Al-Zakwani, Wael Al Mahmeed, Abdullah Shehab, Mohamed Arafah, Ali T. Al-Hinai, Omer Al Tamimi, Mahmoud Al Awadhi, Shorook Al Herz, Faisal Al Anazi, Khalid Al Nemer, Othman Metwally, Akram Alkhadra, Mohammed Fakhry, Hossam Elghetany, Abdel Razak Medani, Afzal Hussein Yusufali, Obaid Al Jassim, Omar Al Hallaq, Fahad Omar Ahmed S. Baslaib, Haitham Amin, Khalid Al-Waili, Khamis Al-Hashmi, Raul D. Santos, Khalid Al-Rasadi

**Affiliations:** 1 Department of Pharmacology & Clinical Pharmacy, College of Medicine & Health Sciences, Sultan Qaboos University, Muscat, Oman; 2 Gulf Health Research, Muscat, Oman; 3 Heart and Vascular Institute-Cleveland Clinic, Abu Dhabi, United Arab Emirates; 4 UAE University, Alain, United Arab Emirates; 5 King Saud University Hospital, Riyadh, Kingdom of Saudi Arabia; 6 Ministry of Health, Muscat, Oman; 7 Hamad Medical Corporation, Doha, Qatar; 8 Al Amiri Hospital, Kuwait City, Kuwait; 9 King Fahad National Guard Hospital, Riyadh, Kingdom of Saudi Arabia; 10 Ministry of Health, Riyadh, Kingdom of Saudi Arabia; 11 School of Medicine, Al-Imam Mohammad Ibn Saud Islamic University (IMSIU), Riyadh, Kingdom of Saudi Arabia; 12 King Fahad General Hospital, Jeddah, Kingdom of Saudi Arabia; 13 King Fahad Hospital of the University, Khobar, Kingdom of Saudi Arabia; 14 Soliman Fakieh Hospital, Jeddah, Kingdom of Saudi Arabia; 15 Dubai Hospital, Dubai, United Arab Emirates; 16 American Hospital, Dubai, United Arab Emirates; 17 Rashid Hospital, Dubai, United Arab Emirates; 18 Bahrain Defense Force Hospital, Manama, Bahrain; 19 Department of Biochemistry, Sultan Qaboos University Hospital, P.O. Box 38, Al-Khod, 123 Muscat, Oman; 20 Department of Physiology, College of Medicine & Health Sciences, Sultan Qaboos University, Muscat, Oman; 21 Lipid Clinic Heart Institute (InCor), University of Sao Paulo Medical School Hospital, Sao Paulo, Brazil

**Keywords:** Metabolic syndrome, Cardiovascular diseases, Triglycerides, Obesity, Blood pressure, HDL cholesterol, LDL cholesterol, Arabian Gulf

## Abstract

**Background:**

The aim of this study was to determine the impact of metabolic syndrome (MetS) on lipid target achievements in the Arabian Gulf.

**Methods:**

The centralized pan-middle east survey on the undertreatment of hypercholesterolemia (CEPHEUS) included 4171 high and very high atherosclerotic cardiovascular disease (ASCVD) risk patients from six Arabian Gulf countries. Analyses were performed using univariate statistics.

**Results:**

The overall mean age was 57 ± 11 years, 41 % were females and 71 % had MetS. MetS patients were less likely to attain their HDL-C (34 vs. 79 %; *P* < 0.001), LDL-C (27 vs. 37 %; *P* < 0.001), non HDL-C (35 vs. 55 %; *P* < 0.001) and Apo B (35 vs. 54 %; *P* < 0.001) compared to those without MetS. Within the MetS cohort, those with very high ASCVD risk were less likely to attain their lipid targets compared to those with high ASCVD risk [HDL-C (32 vs. 41 %; *P* < 0.001), LDL-C (24 vs. 43 %; *P* < 0.001), non HDL-C (32 vs. 51 %; *P* < 0.001) and Apo B (33 vs. 40 %; *P* = 0.001)]. In those with MetS and very high ASCVD risk status, females were less likely to attain their HDL-C (27 vs. 36 %; *P* < 0.001), LDL-C (19 vs. 27 %; *P* < 0.001) and Apo B (30 vs. 35 %; *P* = 0.009) compared to males.

**Conclusions:**

MetS was associated with low lipid therapeutic targets. Women and those with very high ASCVD risk were also less likely to attain their lipid targets in the Arabian Gulf.

## Background

The prevalence of metabolic syndrome (MetS) in the general population is 10–15 % higher in the Arabian Gulf than in most developed countries and is more observed in women (32.1–42.7 %) than men (20.7–37.2 %) [1]. Similarly, MetS is also highly prevalent (46 %) in patients with acute coronary syndrome (ACS) in the Arabian Gulf [2, 3]. MetS is associated with increased risk of developing type 2 diabetes mellitus (T2DM), cardiovascular disease (CVD) and mortality [4, 5]. In the Gulf Registry of Acute Coronary Events (Gulf RACE), which included 8716 consecutive patients hospitalized with ACS in six Arabian Gulf countries, MetS was associated with increased risk for the development of heart failure and recurrent myocardial ischemia without an increase in hospital mortality [2].

The atherogenic dyslipidemia in MetS is characterized by low high-density lipoprotein cholesterol (HDL-C), elevated triglyceride (TG) and increased concentration of small, dense low-density lipoprotein (LDL) particles. Lifestyle therapy to improve atherogenic lipid profile should be recommended to all subjects with MetS [6] and if therapeutic lipid targets are not achieved then maximally tolerated statins or combination therapies should be recommended depending on risk stratification [5].

Despite the high prevalence of MetS and dyslipidemia in the Arabian Gulf region, there are currently no published data assessing the gap in the treatment of dyslipidemia in patients with MetS. Hence, the objective of this study was to evaluate the impact of MetS on lipid target achievements among patients with high and very high atherosclerotic cardiovascular disease (ASCVD) risk status in the Centralized Pan-Middle East Survey on the undertreatment of hypercholesterolemia (CEPHEUS) in the Arabian Gulf.

## Methods

The study has been previously described [7]. Briefly, the CEPHEUS study was a multi-centre non-interventional survey of patients on lipid lowering drugs (LLDs) in six Middle Eastern countries (Saudi Arabia, United Arab Emirates, Oman, Qatar, Bahrain, Kuwait). A total of 5457 patients were enrolled in this survey from outpatient clinics by 177 specialists and primary care physicians. The study was conducted between November 22, 2009 and July 7, 2010. The inclusion criteria were: patients ≥18 years of age; taking LLDs for ≥3 months, with no dose change for a minimum of 6 weeks.

A fasting blood sample was taken from each subject for measurement of total cholesterol (TC), HDL-C, low-density lipoprotein cholesterol (LDL-C), TG, apolipoprotein A1 (Apo A1), apolipoprotein B (Apo B), glucose and glycated haemoglobin A1c (HbA1c). Blood samples were collected in 3 tubes (5 ml in a gel tube, 2 ml in a potassium oxalate/sodium fluoride tube and 2 ml in an ethylenediaminetetra-acetic acid (EDTA) tube. The blood samples were shipped by air courier and the tests were performed at the King Faisal specialist Hospital and Research Centre (Riyadh, Saudi Arabia). All the laboratory tests underwent internal and external quality control checks. Criteria for ASCVD risk status was derived from the National Lipid Association (NLA) recommendations for patient-centered management of dyslipidemia part 1—executive summary [8]. High risk group included patients with ≥3 major ASCVD risk factors, diabetes mellitus (type 1 or 2) with 0–1 other major ASCVD risk factor or LDL-C ≥190 mg/dL (5.02 mmol/L) (severe hypercholesterolemia). Very high risk group included ASCVD and diabetes mellitus with ≥2 other major ASCVD risk factors [8].

As per recent unified definition by the International Diabetes Federation (IDF) and the American Heart Association/National Heart, Lung and Blood Institute (AHA/NHLBI) using the modified National Cholesterol Education Program–Adult Treatment Panel III (NCEP ATP III) guidelines [9], metabolic syndrome was defined as having 3 or more of the following criteria: (1) increased abdominal obesity (waist circumference of ≥94 cm for men and ≥80 cm for women for Middle Eastern (Mediterranean/European) populations), (2) elevated triglycerides of ≥150 mg/dL (1.7 mmol/L), (3) reduced HDL-C of <40 mg/dL (1.0 mmol/L) for males and <50 mg/dL (1.3 mmol/L) for females, (4) elevated BP ≥130 mmHg for systolic and/or ≥85 mmHg for diastolic, and (5) elevated fasting blood glucose of ≥100 mg/dL (5.6 mmol/L).

Therapeutic lipoprotein targets for the high ASCVD risk patients were LDL-C <2.6 mmol/L (100 mg/dL) and LDL-C <1.8 mmol/L (70 mg/dL) for those with high and very high ASCVD risk status, respectively [8]. Blood pressure (BP) goals were adapted from the new Eighth Joint National Committee (JNC-8) 2014 Hypertension Guideline Management Algorithm. BP goals for those without diabetes mellitus (DM) and ≥60 years and those <60 years were <150/90 mmHg and <140/90 mmHg, respectively. For those with DM irrespective of age, the BP goal was <140/90 mmHg [10].

### Statistical analysis

Descriptive statistics were used to describe the data. For categorical variables, frequencies and percentages were reported. Differences between groups were analyzed using Pearson’s χ^2^ tests (or Fisher’s exact tests for cells <5). For continuous variables, mean and standard deviation were used to summarize the data. Analyses were performed using Student’s *t* test. An a priori two-tailed level of significance was set at 0.05. Statistical analyses were conducted using STATA version 13.1 (STATA Corporation, College Station, TX, USA).

### Ethics approval

This study complied with the declaration of Helsinki and had approval from the internal review bodies/ethics committees of each participating institution in each of the Arabian Gulf countries (CEPHEUS; Study Code: SRP-CB-CRE-2006/01). Informed written consent was also obtained from all patients enrolled in the study.

## Results

In total, 5457 patients participated in the survey. However, those that had missing laboratory data, underage (<18 years), missing risk level data as well as those with low and moderate risk were not included in this study. Therefore, the final study sample comprised of 4171 high and very high ASCVD risk patients.

Table 1 outlines the demographics and clinical characteristics of the cohort. The overall mean age of the cohort was 57 ± 11 years with 41 % (n = 1711) females and 77 % (n = 3215) Arab Gulf citizens. The average body mass index (BMI) was 31 ± 7 kg/m^2^. The proportion of patients with coronary heart disease (CHD), diabetes mellitus and hypertension were 36 % (n = 1511), 77 % (n = 3205) and 70 % (n = 2906), respectively. Most of the patients (78 %; n = 3261) had very high ASCVD risk status. Majority (94 %; n = 3928) were on statin monotherapy. Patients on statin combination and other dyslipidemic therapy were 4.8 % (n = 202) and 1.0 % (n = 41), respectively.

**Table 1:** Demographic and clinical characteristics stratified by metabolic syndrome

Characteristic, n (%) unless specified otherwise	All (*N* = 4171)	No MetS (n = 1223) 29 %	Mets (n = 2948) 71 %	*P value*
Gulf citizen	3215 (77 %)	874 (71 %)	2341 (79 %)	<0.001
Female gender	1711 (41 %)	366 (30 %)	1345 (46 %)	<0.001
Age, mean ± SD, years	57 ± 11	57 ± 12	57 ± 11	0.620
Weight, mean ± SD, kg	82 ± 17	78 ± 17	84 ± 17	<0.001
Waist circumference, mean ± SD, cm	104 ± 14	99 ± 14	106 ± 13	<0.001
BMI, mean ± SD, kg/m^2^	31 ± 7	29 ± 6	32 ± 7	<0.001
BMI >30 kg/m^2^	2219 (53 %)	484 (40 %)	1735 (59 %)	<0.001
Current smoker	517 (12 %)	168 (14 %)	349 (12 %)	0.090
Hypertension	2906 (70 %)	750 (61 %)	2156 (73 %)	<0.001
Coronary heart disease	1511 (36 %)	554 (45 %)	957 (32 %)	<0.001
Peripheral vascular disease	142 (3.4 %)	52 (4.3 %)	90 (3.1 %)	0.052
Cerebrovascular disease	183 (4.4 %)	53 (4.3 %)	130 (4.4 %)	0.913
Diabetes mellitus	3205 (77 %)	768 (63 %)	2437 (83 %)	<0.001
HbA1c, mean ± SD, %	8.62 ± 3.79	7.84 ± 2.61	8.88 ± 4.06	<0.001
HbA1c <7 %	820 (26 %)	320 (42 %)	500 (21 %)	<0.001
*ASCVD risk status*				
High risk	910 (22 %)	335 (27 %)	575 (20 %)	<0.001
Very high risk	3261 (78 %)	888 (73 %)	2373 (81 %)	<0.001
*Dyslipidaemic therapy*				
Statin monotherapy	3928 (94 %)	1146 (94 %)	2782 (94 %)	0.218
Statin combination	202 (4.8 %)	67 (5.5 %)	135 (4.6 %)	0.486
Others	41 (1.0 %)	10 (0.8 %)	31 (1.1 %)	0.404
*Lipid levels on treatment, mean* *±* *SD, mmol/L, unless specified otherwise*				
TC	4.31 ± 1.11	4.15 ± 1.08	4.38 ± 1.12	<0.001
LDL-C	2.54 ± 0.94	2.43 ± 0.95	2.58 ± 0.93	<0.001
HDL-C	1.15 ± 0.31	1.30 ± 0.32	1.10 ± 0.28	<0.001
Apo B, g/L	0.92 ± 0.27	0.84 ± 0.26	0.95 ± 0.27	<0.001
Non-HDL-C	3.16 ± 1.09	2.85 ± 1.01	3.28 ± 1.09	<0.001
TG	1.75 ± 1.27	1.19 ± 0.46	1.98 ± 1.42	<0.001
*Lipid goal attainments, n (%)*				
HDL-C goal	1952 (47 %)	963 (79 %)	989 (34 %)	<0.001
LDL-C goal	1258 (30 %)	449 (37 %)	809 (27 %)	<0.001
Apo B goal	1676 (40 %)	658 (54 %)	1018 (35 %)	<0.001
Non-HDL-C goal	1715 (41 %)	673 (55 %)	1042 (35 %)	<0.001
*Blood pressure (BP), mean* *±* *SD unless specified otherwise*				
Systolic BP, mmHg	133 ± 19	125 ± 17	136 ± 18	<0.001
Diastolic BP, mmHg	79 ± 10	75 ± 10	80 ± 10	<0.001
BP control, n (%)	2497 (60 %)	957 (78 %)	1540 (52 %)	<0.001

As per recent unified definition by the International Diabetes Federation (IDF) and the American Heart Association/National Heart, Lung and Blood Institute (AHA/NHLBI) using the modified National Cholesterol Education Program–Adult Treatment Panel III (NCEP ATP III) guidelines, MetS was defined as having 3 or more of the following criteria: (1) increased abdominal obesity (waist circumference of ≥94 cm for men and ≥80 cm for women for Middle Eastern (Mediterranean/European) populations), (2) elevated triglycerides of ≥150 mg/dL (1.7 mmol/L), (3) reduced HDL-C of <40 mg/dL (1.04 mmol/L) for males and <50 mg/dL (1.3 mmol/L) for females, (4) elevated BP ≥130 mmHg for systolic and/or ≥85 mmHg for diastolic, and (5) elevated fasting blood glucose of ≥100 mg/dL (5.6 mmol/L)

Criteria for ASCVD risk status was adapted from the National Lipid Association criteria for atherosclerotic cardiovascular disease. High risk group included patients with ≥3 major ASCVD risk factors, diabetes mellitus (type 1 or 2) with 0/1 major ASCVD risk factor and LDL-C ≥190 mg/dL (5.02 mmol/L) (severe hypercholesterolemia). Very high risk group included ASCVD (CHD, PAD, CVD), diabetes mellitus with ≥2 other major ASCVD risk factors

Despite the lack of a recommended HDL-C goal by guidelines, satisfactory HDL-C was defined as <40 mg/dL (1.04 mmol/L) for males or <50 mg/dL (1.3 mmol/L) for females. Therapeutic lipoprotein targets for the high risk patients were LDL-C <2.6 mmol/L, apo B <0.90 g/L and non-HDL-C <3.3 mmol/L. For the highest risk group therapeutic lipoprotein targets were LDL-C <1.8 mmol/L, apo B <0.80 g/L and non-HDL-C <2.6 mmol/L

BP goals were adapted from the new JNC-8 2014 Hypertension Guideline Management Algorithm. BP goals for those without diabetes mellitus (DM) and ≥60 years and those <60 years were <150/90 mmHg and <140/90 mmHg, respectively. For those with DM irrespective of age, the BP goal was <140/90 mmHg

*MetS* metabolic syndrome, *SD* standard deviation, *BMI* body mass index, *CHD* coronary heart disease, *PAD* peripheral arterial disease, *CVD* cardiovascular disease, *ASCVD* atherosclerotic cardiovascular disease, *TC* total cholesterol, *LDL*-*C* low-density lipoprotein cholesterol, *HDL*-*C* high-density lipoprotein cholesterol, *Apo B* apolipoprotein B, *TG* triglyceride

MetS patients were more likely to be female (46 vs. 30 %; *P* < 0.001), associated with higher waist circumference (106 vs. 99 cm; *P* < 0.001) and BMI (32 vs. 29 kg/m^2^; *P* < 0.001), hypertensive (73 vs. 62 %; *P* < 0.001), diabetic (83 vs. 63 %; *P* < 0.001) and very high ASCVD risk status (81 vs. 73 %; *P* < 0.001). MetS patients were also less likely to attain HDL-C (34 vs. 79 %; *P* < 0.001), LDL-C (27 vs. 37 %; *P* < 0.001), Apo B (35 vs. 54 %; *P* < 0.001) and non HDL-C (35 vs. 55 %; *P* < 0.001) lipid targets.

**Fig. 1:**
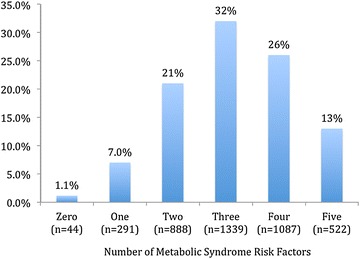
Number of metabolic syndrome (MetS) risk factors (increased abdominal obesity, elevated triglycerides, reduced HDL-C, elevated blood pressure, and elevated blood glucose) in atherosclerotic cardiovascular disease (ASCVD) risk patients (*N* = 4171). As per recent unified definition by the International Diabetes Federation (IDF) and the American Heart Association/National Heart, Lung and Blood Institute (AHA/NHLBI) using the modified National Cholesterol Education Program–Adult Treatment Panel III (NCEP ATP III) guidelines, MetS was defined as having three or more of the following criteria: (1) increased abdominal obesity (waist circumference of ≥94 cm for men and ≥80 cm for women for Middle Eastern (Mediterranean/European) populations), (2) elevated triglycerides of ≥150 mg/dL (1.7 mmol/L), (3) reduced HDL-C of <40 mg/dL (1.04 mmol/L) for males and <50 mg/dL (1.3 mmol/L) for females, (4) elevated BP ≥130 mmHg for systolic and/or ≥85 mmHg for diastolic, and (5) elevated fasting blood glucose of ≥100 mg/dL (5.6 mmol/L). Criteria for ASCVD risk status was adapted from the National Lipid Association criteria for atherosclerotic cardiovascular disease. High risk group included patients with ≥3 major ASCVD risk factors, diabetes mellitus (type 1 or 2) with 0/1 major ASCVD risk factor and LDL-C ≥190 mg/dL (5.02 mmol/L) (severe hypercholesterolemia). Very high risk group included ASCVD (CHD, PAD, CVD), diabetes mellitus with ≥2 other major ASCVD risk factors

**Fig. 2:**
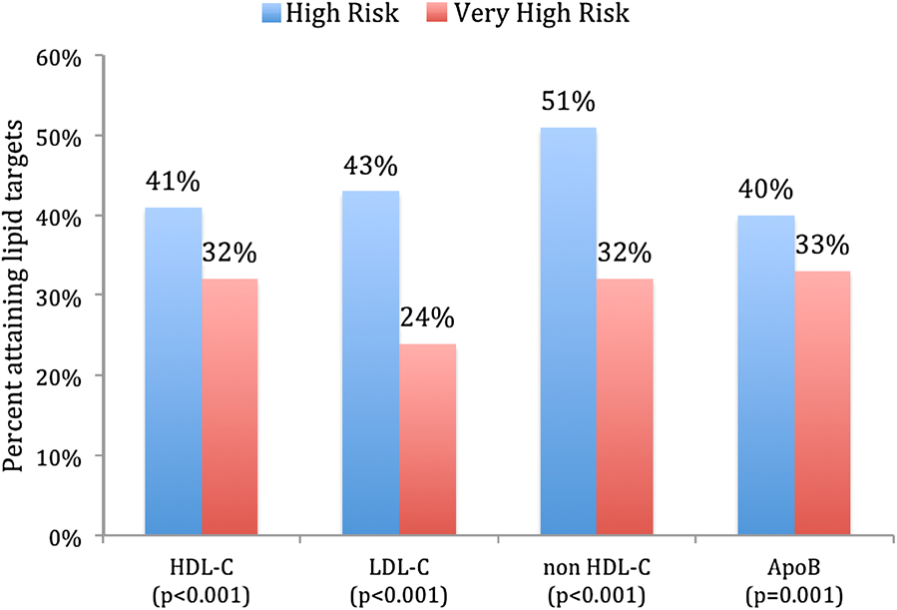
Lipid target achievements (HDL-C, LDL-C, non HDL-C and Apo B) in metabolic syndrome patients stratified by atherosclerotic cardiovascular disease (ASCVD) risk status (*N* = 2948). *HDL*-*C* high-density lipoprotein cholesterol, *LDL*-*C* low-density lipoprotein cholesterol, *Apo B* apolipoprotein B. As per recent unified definition by the International Diabetes Federation (IDF) and the American Heart Association/National Heart, Lung and Blood Institute (AHA/NHLBI) using the modified National Cholesterol Education Program–Adult Treatment Panel III (NCEP ATP III) guidelines, MetS was defined as having three or more of the following criteria: (1) increased abdominal obesity (waist circumference of ≥94 cm for men and ≥80 cm for women for middle eastern (Mediterranean/European) populations), (2) elevated triglycerides of ≥150 mg/dL (1.7 mmol/L), (3) reduced HDL-C of <40 mg/dL (1.0 mmol/L) for males and <50 mg/dL (1.3 mmol/L) for females, (4) elevated BP ≥130 mmHg for systolic and/or ≥85 mmHg for diastolic, and (5) elevated fasting blood glucose of ≥100 mg/dL (5.6 mmol/L). Criteria for ASCVD risk status was adapted from the National Lipid Association criteria for atherosclerotic cardiovascular disease. High risk group included patients with ≥3 major ASCVD risk factors, diabetes mellitus (type 1 or 2) with 0–1 major ASCVD risk factors, LDL-C ≥190 mg/dL (severe hypercholesterolemia). Very high risk group included ASCVD (CHD, PAD, CVD), diabetes mellitus with ≥2 other major ASCVD risk factors. Despite the lack of a recommended HDL-C goal by guidelines, satisfactory HDL-C was defined as <40 mg/dL (1.03 mmol/L) for males or <50 mg/dL (1.29 mmol/L) for females. Therapeutic lipoprotein targets for the highest risk group were LDL-C <1.8 mmol/L, apo B <0.80 g/L and non-HDL-C <2.6 mmol/L

Figure 1 shows that the number of patients with 1, 2, 3, 4 and 5 MetS risk factors were 7.0 % (n = 291), 21 % (n = 888), 32 % (n = 1339), 26 % (n = 1087) and 13 % (n = 522), respectively. Figure 2 outlines the lipid target achievements (HDL-C, LDL-C, non HDL-C and Apo B) in metabolic syndrome patients stratified by ASCVD risk status. MetS patients with very high ASCVD risk status were less likely to attain HDL-C (32 vs. 41 %; *P* < 0.001), LDL-C (24 vs. 43 %; *P* < 0.001), non HDL-C (32 vs. 51 %; *P* < 0.001) and Apo B (33 vs. 40 %; *P* = 0.001) lipid targets when compared to those with high ASCVD risk status.

**Fig. 3:**
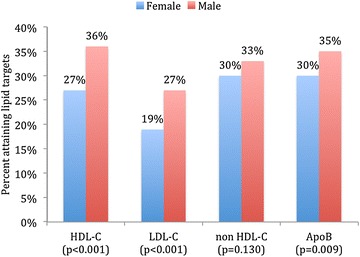
Lipid target achievements (HDL-C, LDL-C, non HDL-C and Apo B) in patients with metabolic syndrome and very high atherosclerotic cardiovascular disease (ASCVD) risk status stratified by gender (*N* = 2373). *HDL*-*C* high-density lipoprotein cholesterol, *LDL*-*C* low-density lipoprotein cholesterol, *Apo B*, apolipoprotein B. As per recent unified definition by the International Diabetes Federation (IDF) and the American Heart Association/National Heart, Lung and Blood Institute (AHA/NHLBI) using the modified National Cholesterol Education Program–Adult Treatment Panel III (NCEP ATP III) guidelines, MetS was defined as having 3 or more of the following criteria: (1) increased abdominal obesity (waist circumference of ≥94 cm for men and ≥80 cm for women for Middle Eastern (Mediterranean/European) populations), (2) elevated triglycerides of ≥150 mg/dL (1.7 mmol/L), (3) reduced HDL-C of <40 mg/dL (1.0 mmol/L) for males and <50 mg/dL (1.3 mmol/L) for females, (4) elevated BP ≥130 mmHg for systolic and/or ≥85 mmHg for diastolic, and (5) elevated fasting blood glucose of ≥100 mg/dL (5.6 mmol/L). Criteria for ASCVD risk status was adapted from the National Lipid Association criteria for atherosclerotic cardiovascular disease. High risk group included patients with ≥3 major ASCVD risk factors, diabetes mellitus (type 1 or 2) with 0–1 major ASCVD risk factors, LDL-C ≥190 mg/dL (severe hypercholesterolemia). Very high risk group included ASCVD (CHD, PAD, CVD), diabetes mellitus with ≥2 other major ASCVD risk factors. Despite the lack of a recommended HDL-C goal by guidelines, satisfactory HDL-C was defined as <40 mg/dL (1.03 mmol/L) for males or <50 mg/dL (1.29 mmol/L) for females. Therapeutic lipoprotein targets for the highest risk group were LDL-C <1.8 mmol/L, apo B <0.80 g/L and non-HDL-C <2.6 mmol/L

**Fig. 4:**
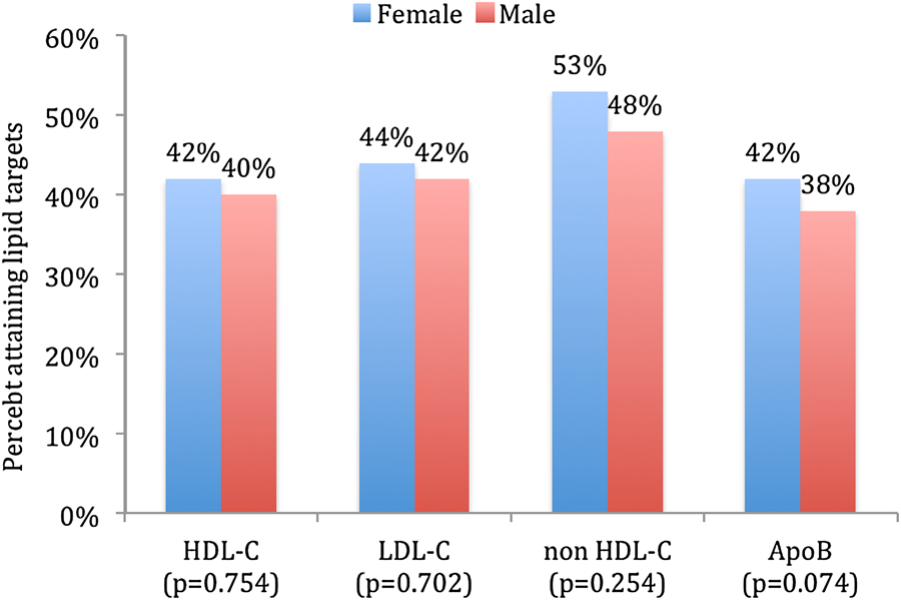
Lipid target achievements (HDL-C, LDL-C, non HDL-C and Apo B) in patients with metabolic syndrome and high atherosclerotic cardiovascular disease (ASCVD) risk status stratified by gender (*N* = 575). *HDL*-*C* high-density lipoprotein cholesterol, *LDL*-*C* low-density lipoprotein cholesterol, *Apo B* apolipoprotein B. As per recent unified definition by the International Diabetes Federation (IDF) and the American Heart Association/National Heart, Lung and Blood Institute (AHA/NHLBI) using the modified National Cholesterol Education Program–Adult Treatment Panel III (NCEP ATP III) guidelines, MetS was defined as having 3 or more of the following criteria: (1) increased abdominal obesity (waist circumference of ≥94 cm for men and ≥80 cm for women for Middle Eastern Mediterranean/European) populations), (2) elevated triglycerides of ≥150 mg/dL (1.7 mmol/L), (3) reduced HDL-C of <40 mg/dL (1.0 mmol/L) for males and <50 mg/dL (1.3 mmol/L) for females, (4) elevated BP ≥130 mmHg for systolic and/or ≥85 mmHg for diastolic, and (5) elevated fasting blood glucose of ≥100 mg/dL (5.6 mmol/L). Criteria for ASCVD risk status was adapted from the National Lipid Association criteria for atherosclerotic cardiovascular disease. High risk group included patients with ≥3 major ASCVD risk factors, diabetes mellitus (type 1 or 2) with 0–1 major ASCVD risk factors, LDL-C ≥190 mg/dL (severe hypercholesterolemia). Very high risk group included ASCVD (CHD, PAD, CVD), diabetes mellitus with ≥2 other major ASCVD risk factors. Despite the lack of a recommended HDL-C goal by guidelines, satisfactory HDL-C was defined as <40 mg/dL (1.03 mmol/L) for males or <50 mg/dL (1.29 mmol/L) for females. Therapeutic lipoprotein targets for the high risk patients were LDL-C <2.6 mmol/L, apo B <0.90 g/L and non-HDL-C <3.3 mmol/L

In MetS patients with very high ASCVD risk status (Fig. 3), females were less likely to attain HDL-C (27 vs. 36 %; *P* < 0.001), LDL-C (19 vs. 27 %; *P* < 0.001) and Apo B (30 vs. 35 %; *P* = 0.009) lipid targets when compared to males. However, there were no significant differences in lipid target achievements between genders in MetS patients with high ASCDV risk status (Fig. 4).

## Discussion

To our best knowledge this the first study to assess the lipid attainment goals in patients with MetS in the Arabian Gulf. The prevalence of MetS was 71 % in patients on LLDs in the Arabian Gulf. MetS was more prevalent in the Gulf citizens, females and patients with very high ASCVD risk status. Patients with MetS were significantly less likely to attain their LDL-C (27 vs. 37 %; *P* < 0.001), non HDL-C (35 vs. 55 %; *P* < 0.001) and apo B (35 vs. 54 %; *P* < 0.001) targets compared to patients without MetS.

MetS is defined as a cluster of cardiovascular risk factors including central obesity, raised serum TG, reduced HDL-C, glucose intolerance, and hypertension [5, 9]. Additional abnormalities like the pro-inflammatory and pro-thrombotic factors are considered part of the constellation of risk factors in MetS [5, 11], that were not measured in the current study. There are several guideline definitions of MetS [12], The calculation of the MetS prevalence in the current study was based on the harmonized definition developed by the a joint statement of the International Diabetes Federation (IDF) Task Force on Epidemiology and Prevention, National Heart, Lung and Blood Institute⁄American Heart Association (NHLBI⁄AHA), the World Heart Federation, the International Atherosclerosis Society, and the International Association for the Study of Obesity [9].

Insulin resistance plays a major role in lipid derangement in patients with MetS, which is characterized by both quantitative dyslipidemia (high TG and low HDL-C) and qualitative dyslipidemia (small, dense, apo B-100-rich LDL). These phenotypes of atherogenic dyslipidemia in the presence or absence of increased levels of LDL-C is the most frequent dyslipidemia observed in patients with MetS and are strongly associated with atherosclerosis and premature coronary artery disease (CAD) [12–18]. In insulin resistance, there is an increase in free fatty acids (FFAs) flux to the liver that stimulate the synthesis of very low density lipoprotein (VLDL) particles and results in high TG levels and Apo B particles in plasma. Insulin resistance can also impair the lipolysis of VLDL particles that leads to an accumulation of triglyceride-rich remnant lipoproteins (VLDL-remnants) and subsequent transfer of cholesterol esters in exchange for triglycerides from the HDL particles to the triglyceride-rich remnant through the action of cholesterol ester transfer protein (CETP). This will results in smaller HDL particles and low levels of HDL-C [19–23]. Moreover, insulin resistance is associated with an increase in C-reactive protein (CRP), a marker of inflammation which has been shown to increase linearly with the number of metabolic syndrome components present [24], associated with a higher risk of developing diabetes [25, 26] and CAD [27].

MetS identifies people at a higher risk of CVD and diabetes than the general population. MetS is a five-fold increased risk of diabetes, a two-fold increase in cardiovascular outcomes and 1.5-fold increase in all-cause mortality according to the recent meta-analysis [4, 16, 28]. In our study, 32 % of the MetS cohort carried three of the major cardiovascular risk factors. Moreover, MetS was observed in 81 % of patients with very high ASCVD compared to 20 % of high ASCVD risk status. Therefore, this highlights the high cardiovascular risk profile of our patients and the need for more intensive risk factors stratification and treatment in this population.

The management of MetS should focus on reducing both short and long term risk of developing subsequent cardiovascular events. The treatment should focus on optimal controlling of the various components of MetS. Life style modifications through weight loss, diet and exercise [6] should be the first line intervention in patients with MetS. However, in patients with high, very high ASCVD risk and in patients who fail life style intervention pharmacological therapies should be considered to control atherogenic dyslipidemia [8, 29, 30], diabetes [31], hypertension [10] and current guidelines for their management should be followed. For the treatment of atherogenic dyslipidemia, statins are recommended as the first line therapy. Although currently there are no clinical trials addressing the cardiovascular outcomes of LLDs on patients with the MetS, nevertheless, in the subgroup of patients with MetS, clinical trials showed benefits from LLDs particularly statins on reducing cardiovascular events that is considered similar or greater, compared with overall study populations [32–35].

Moreover, in the Treating to New Target study (TNT) trial, in the subgroup of patients with MetS, CAD and no diabetes there was a significant additional benefit on cardiovascular events when high dose atorvastatin of 80 mg was used compared to the low atorvastatin dose of 10 mg [32]. The use of combination therapy to optimize other lipid targets beyond LDL-C like non HDL-C, Apo B, TG and HDL-C may be considered in treating atherogenic dyslipidemia in MetS. Non HDL-C is considered to be a better predictor of CV risk and therapeutic target than LDL-C, particularly in patients with diabetes and MetS, and therefore the NLA in their recent guideline, have placed non HDL-C ahead of LDL-C as a therapeutic target [8]. Apart from adding ezetimibe to statins for combination therapy [36], other combination therapies like fibrates and niacin [37] have failed to show additional benefits in reducing cardiovascular events. Nonetheless, adding fibrates particularly fenofibrate to statins, have proven to be safe and effective in reducing cardiovascular events in subgroup of patients with obesity, high TG and low HDL-C [38, 39] and therefore, should be considered to treat high risk patients with atherogenic dyslipidemia when high dose statins failed to achieve the lipid therapeutic targets.

In our study MetS patients with very high ASCVD risk status were less likely to attain LDL-C (24 vs. 43 %; *P* < 0.001) and non HDL-C (32 vs. 51 %; *P* < 0.001) lipid targets when compared with those with high ASCVD risk status. Compared to the Dyslipidemia International Study of China (DYSIS-China), which was an observational study of 25,697 patients, in which 37 % had CAD, 57 % had diabetes and over one-third had MetS. LDL-C goal was achieved in 47 % of patients with MetS compared to 69 % in those without the MetS (*P* < 0.001). Non HDL-C goals were achieved in 51 % of patients with MetS compared to 72 % in those without the MetS (*P* < 0.001). Among very high-risk individuals, only 26 % achieved their LDL-C goal and 42 % attained their non HDL-C goal [40]. Similar to the DYSIS-China study, the current study showed that the majority of MetS patients were on statin therapy (94 %), the use of other non-statin (1.1 %) or combination therapies (4.6 %) were low [7]. Therefore, the recommendation from both studies is to increase the awareness of both patients and clinicians regarding the atherogenic dyslipidemia and increased ASCVD risk associated with the MetS. In order to improve therapeutic lipid goals attainment and reduce ASCVD risk in MetS, effective strategies need to be developed and implemented and should primarily focused on life style modifications and to use more intensive LLDs and combination therapies in patients with very-high risk ASCVD risk status.

In a systematic review conducted by Mabry and colleagues, the reported prevalence of the MetS in the general population in the Arabian Gulf is 10–15 % higher than in most developed countries and it is higher in women (32.1–42.7 %) than men (20.7–37.2 %) [1]. There was no report concerning statin use and the attainment of lipid targets in this systematic review [1]. We observed similar higher prevalence of MetS in women than men (46 vs. 30 %; *P* < 0.001). Moreover; in our study females with MetS and very high ASCVD risk status were less likely to attain lipid targets when compared to males. The gap in lipid goals between men and women in our study may be explained by the high prevalence of T2DM and MetS in women compared to men. In addition, these findings may be associated with other factors like baseline lipid levels, socioeconomic status, marital status and the dosage of statin [41–43]. The prevalence of MetS in our study (71 %) was higher than that seen in the Gulf RACE study (46 %), in patients with ACS in the Arabian Gulf. In this study they observed higher statin usage among patients with MetS compared to patients with no MetS (83 vs. 79 %) but there was no report concerning the attainment of lipid targets [2, 3]. The difference in the prevalence of metabolic syndrome between these studies can be explained by factors like the type of the population understudy, the number of cardiovascular co-morbidities and other associated risk factors and the difference in the criteria used to define MetS.

Our findings provide a useful overview of current lipid management and treatment outcomes in patients with MetS in the Middle East. However, they study is not without limitations; it is an observational cross-sectional trial that did not assess long-term outcomes. A prospective follow-up study is required to evaluate medical treatment and attainment in relation to mortality in patients treated with LLDs. The population studied is relatively small and considerable variability in practice patterns across the Arabian Gulf exists, and probably even among study sites, and therefore caution should be exercised when extrapolating the results to the general population. In addition, the population included only patients who were already on LLDs. It was not clear what proportion of eligible patients with appropriate risk factors were offered LLDs in different countries. Such information would be important to evaluate the overall burden of the disease among untreated patients in various countries.

## Conclusion

This study has demonstrated that MetS is highly prevalent and is associated with low attainment of lipid therapeutic targets in the Arabian Gulf. Furthermore, women and those with very high ASCVD risk were also less likely to attain their lipid targets. In order to improve therapeutic lipid goal attainment and reduce ASCVD risk in MetS in the Arabian Gulf region, effective strategies need to be developed and implemented and should primarily be focused on life style modifications and more intensive LLDs and combination therapies in patients with high risk ASCVD risk status.

## Abbreviations

CEPHEUS: centralized pan-middle east survey on the undertreatment of hypercholesterolemia
MetS: metabolic syndrome
ASCVD: atherosclerotic cardiovascular disease
HDL-C: high-density lipoprotein cholesterol
LDL-C: low-density lipoprotein cholesterol
Apo B: apolipoprotein B
ACS: acute coronary syndrome
Gulf RACE: Gulf Registry of Acute Coronary Events
TG: triglyceride
LLD: lipid lowering drugs
TC: total cholesterol
Apo A1: apolipoprotein A1
HbA1c: glycated hemoglobin
EDTA: ethylenediaminetetra-acetic acid
NLA: National Lipid Association
IDF: International Diabetes Federation
AHA/NHLBI: American Heart Association/National Heart, Lung and Blood Institute
NCEP ATP III: National Cholesterol Education Program–Adult Treatment Panel III
JNC-8: Eighth Joint National Committee
BP: blood pressure
DM: diabetes mellitus
BMI: body mass index
CHD: coronary heart disease
CAD: coronary artery disease
FFAs: free fatty acids
VLDL: very low density lipoprotein
CETP: cholesterol ester transfer protein
CRP: C-reactive protein
TNT: treating to new target study
